# MEHunter: transformer-based mobile element variant detection from long reads

**DOI:** 10.1093/bioinformatics/btae557

**Published:** 2024-09-16

**Authors:** Tao Jiang, Zuji Zhou, Zhendong Zhang, Shuqi Cao, Yadong Wang, Yadong Liu

**Affiliations:** Center for Bioinformatics, Faculty of Computing, Harbin Institute of Technology, Harbin, Heilongjiang 150001, China; Zhengzhou Research Institute, Harbin Institute of Technology, Zhengzhou, Henan 450000, China; Center for Bioinformatics, Faculty of Computing, Harbin Institute of Technology, Harbin, Heilongjiang 150001, China; Center for Bioinformatics, Faculty of Computing, Harbin Institute of Technology, Harbin, Heilongjiang 150001, China; Center for Bioinformatics, Faculty of Computing, Harbin Institute of Technology, Harbin, Heilongjiang 150001, China; Center for Bioinformatics, Faculty of Computing, Harbin Institute of Technology, Harbin, Heilongjiang 150001, China; Zhengzhou Research Institute, Harbin Institute of Technology, Zhengzhou, Henan 450000, China; Center for Bioinformatics, Faculty of Computing, Harbin Institute of Technology, Harbin, Heilongjiang 150001, China; Zhengzhou Research Institute, Harbin Institute of Technology, Zhengzhou, Henan 450000, China

## Abstract

**Summary:**

Mobile genetic elements (MEs) are heritable mutagens that significantly contribute to genetic diseases. The advent of long-read sequencing technologies, capable of resolving large DNA fragments, offers promising prospects for the comprehensive detection of ME variants (MEVs). However, achieving high precision while maintaining recall performance remains challenging mainly brought by the variable length and similar content of MEV signatures, which are often obscured by the noise in long reads. Here, we propose MEHunter, a high-performance MEV detection approach utilizing a fine-tuned transformer model adept at identifying potential MEVs with fragmented features. Benchmark experiments on both simulated and real datasets demonstrate that MEHunter consistently achieves higher accuracy and sensitivity than the state-of-the-art tools. Furthermore, it is capable of detecting novel potentially individual-specific MEVs that have been overlooked in published population projects.

**Availability and implementation:**

MEHunter is available from https://github.com/120L021101/MEHunter.

## 1 Introduction

Mobile genetic element variants (MEVs) account for approximately 25% of structural variations (SVs) in the human genome (Gardner et al. 2017), encompassing elements such as long interspersed nuclear element 1 (L1), Alu, and SINE-VNTR-Alu (SVA) elements. Active MEVs act as insertional mutagens that can alter genetic traits, potentially disrupting gene function and leading to various genetic disorders (Kojima et al. 2023).

Long-read sequencing technologies represent a significant advancement over traditional next-generation sequencing (NGS) by offering extended sequence lengths. This capability enhances genome-spanning ability, thereby providing a detailed resolution of SVs across a broad spectrum of scales and types (Porubsky and Eichler 2024). Characterized by their variable lengths and often only partial or fragmented sequence components, MEVs present a particular challenge for detection. Current algorithms such as rMETL (Jiang et al. 2019), Palmer (Zhou et al. 2020), and xTea (Chu et al. 2021), while effective in some contexts, still occasionally struggle due to their inadequate parsing of nuanced sequence content crucial for accurately identifying MEVs. This underscores the need for more refined detection methods capable of addressing the complex nature of MEVs.

Herein, we introduce MEHunter, an innovative long-read-based mobile element insertions and deletions (MEIs/MEDs) detection tool through fine-tuned transformer model. MEHunter not only enhances the accuracy of MEV detection but also provides researchers with the flexibility to focus on specific MEV events according to their study objectives. MEHunter represents a significant leap forward in the detection of MEVs, promising to unlock new possibilities in genomic and clinical studies.

## 2 Materials and methods

MEHunter identifies MEVs through the following four steps.

- MEHunter utilizes a modified version of cuteSV (Jiang et al. 2020) to precisely and exhaustively identify generic SV characteristics (e.g. loci, signatures, genotypes, etc) from the Binary Alignment Map (BAM) files;
- MEHunter clusters the extracted features of the SVs and employs abPOA (pyabpoa v1.4.3) (Gao et al. 2021) to build the consensus sequence for each cluster;
- MEHunter uses the consensus sequences along with known ME sequences as inputs for a lightweight, modified Smith-Waterman (SW) algorithm to achieve first-round MEVs classification;
- For the remaining unclassified consensus sequences, MEHunter uses minimap2 (Li 2018) as a preclassifier to exclude completely unrelated sequences and applies fine-tuned DNABERT2 (Zhou et al. 2023) to enhance the detection of potential MEVs.

Please also refer to Supplementary Figs S1 and S2 for schematic illustrations and consult Supplementary Notes for more detailed information on the implementation of MEHunter.

## 3 Results and discussion

To evaluate the performance in identifying MEVs, we conducted a comprehensive comparative analysis of MEHunter, rMETL (v1.0), Palmer (v2.0.0, termed as Palmer2), and xTea (v0.1.0) to assess their performance in detecting MEVs on both simulated and real long-read datasets. Palmer2 was excluded from the comparison due to its relatively lower computational efficiency. Moreover, it consistently failed to report the MEVs of the SVA class, as the program crashed.

### 3.1 Assessment on simulated datasets

PacBio HiFi-like and Oxford Nanopore Technologies (ONT)-like long-read sequencing datasets at four sequencing depths (5×, 10×, 20×, and 30×) were simulated using an in silico diploid human genome. The genome includes 20 000 MEVs comprising Alu, SVA, and L1 elements, alongside 5000 ordinary SVs, as detailed in Section 2.1 of the Supplementary Notes. For MEHunter, rMETL, Palmer2, and xTea, default parameters were utilized to call MEVs, except for adjustments to the number of supporting reads (specified by the -s parameter), as outlined in Supplementary Tables S1 and S2.

**Figure 1.**
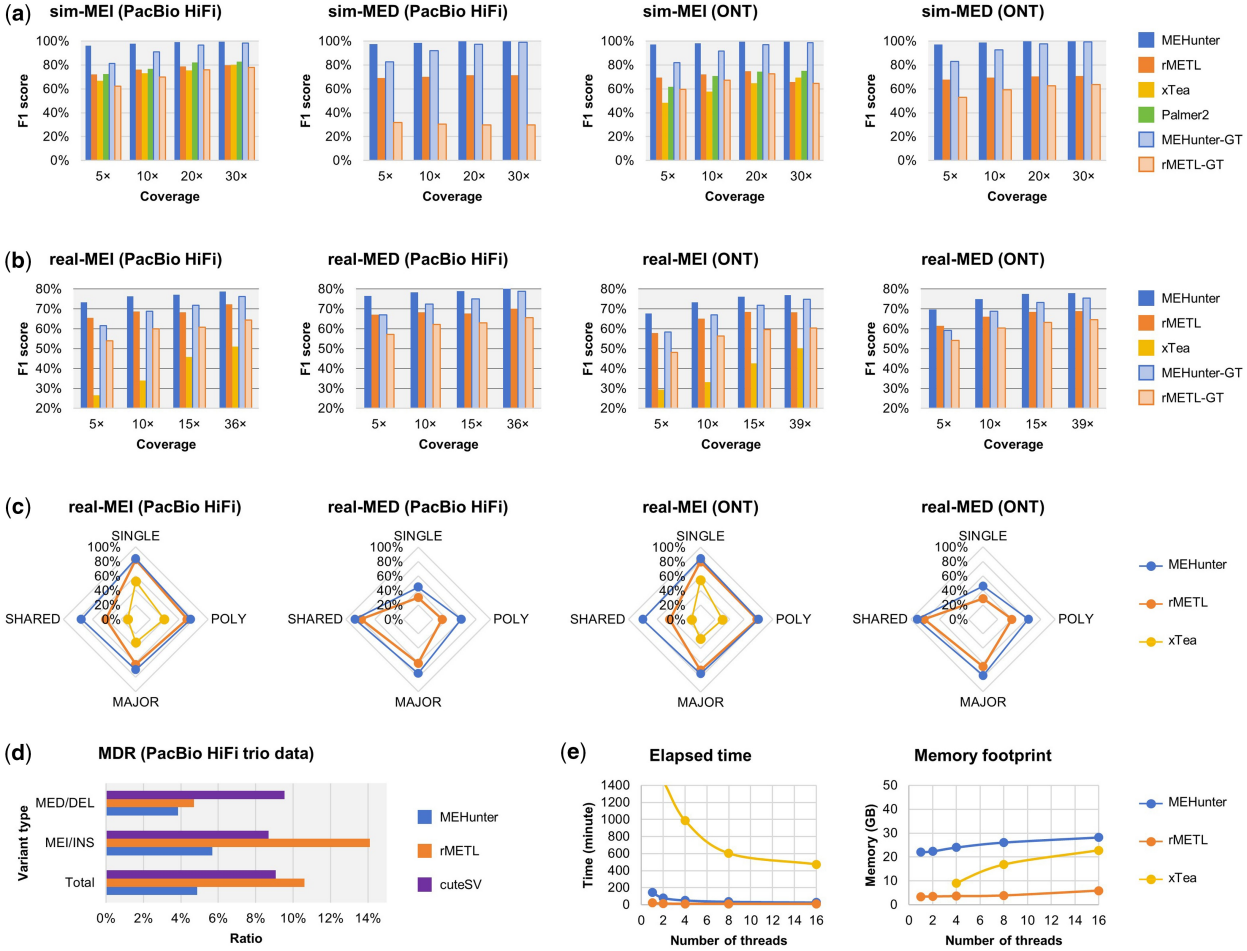
Benchmarking the performance of MEV detection on simulated (sim-) and real (real-) long-read sequencing data. (a) Evaluations across varying coverages of simulated PacBio HiFi and ONT long reads. (b) Evaluations across different coverages of authentic PacBio HiFi and ONT long reads for the HG00731 human individual. (c) Distribution of MEV identification rates for the HG00731 sample, categorized by the presence of SVs shared among 32 individuals. (d) Benchmark results of MDRs for trio data (HG00731, HG00732, and HG00733). (e) Benchmarking results for elapsed time and memory footprint using 15× HG00731 PacBio HiFi data. In the figure, “N” and “N-GT” indicate the statistics without and with genotyping, respectively

Overall, MEHunter exhibited exceptional performance, achieving F1 scores exceeding 99.42% for both MEIs and MEDs across various 30× sequencing datasets (as shown in Fig. 1a and detailed in Supplementary Tables S1 and S2). The performance marks at least a 16.48% improvement over the scores attained by rMETL, Palmer2, and xTea. Notably, MEHunter maintained consistent genotyping accuracy with F1 scores surpassing 98.43%, which significantly highlights its superior capabilities. Moreover, MEHunter obtained the lowest false discovery rates (FDRs) on the simulated datasets. It is also worth noting that Palmer2 and xTea only detect MEIs and cannot report the corresponding genotypes.

Furthermore, it is well-documented that the ability to detect MEVs diminishes with reduced sequencing depth. However, MEHunter exhibits a significantly milder decline in performance compared to the other three tools. For example, in terms of MEV presence/genotype, MEHunter’s performance drops from 99%/98% to 96%/81% on average, whereas the other tools like rMETL averagely decreases from 80%/67% to 68%/46% and lower. Impressively, even at a low sequencing depth of 5×, MEHunter still achieves F1 scores of over 96% for MEV presence and 81% for genotype consistency. These exemplary performances are attributed primarily to MEHunter’s exquisite sensitivity in detecting all types of MEVs.

### 3.2 Assessment of HG00731 human sample

Considering that simulation techniques inherently suffer from limitations in accurately representing the full breadth of complexities present in real-world scenarios, MEHunter, rMETL, and xTea were further benchmarked on the well-studied human sample HG00731. This sample includes 36× real PacBio HiFi data and 39× real ONT data. A callset proposed by the HGSVC2 Project (Ebert et al. 2021), which constructs MEVs through haplotype-resolved SV calling on 32 unrelated human individuals, was employed as a pseudo-ground truth.

Similar to the results of simulated data, MEHunter consistently outperformed rMETL and xTea in detecting MEVs across various sequencing platforms and depths, achieving F1 scores that were 5%–14% higher for both presence and genotype consistency, and reporting more consistent MEVs on PacBio and ONT platforms simultaneously. These results are detailed in Fig. 1b, Supplementary Fig. S3, and Supplementary Tables S3 and S4. In terms of FDR, MEHunter achieved the lowest FDR in most cases, although it fell behind xTea in some instances. However, given xTea’s extremely limited detection capability and its focus solely on detecting MEI presence, MEHunter remains the more powerful tool overall. Additionally, we conducted an ablation study to ascertain the impact of the transformer model on MEV detection. This study compared MEHunter’s performance with and without the minimap2-DNABERT2 module, referred to as MEHunter-nDL. Significantly, incorporating the transformer model enhanced sensitivity by about 40%, enabling MEHunter to achieve approximately a 40% increase in F1 scores in various scenarios. A representative example of this improvement is shown in Supplementary Fig. S4, highlighting MEVs that were missed by MEHunter-nDL, rMETL, and xTea but accurately detected by MEHunter.

Subsequently, we meticulously examine the MEV identification rates across different variant allele frequency (AF) groups with the MEV callsets derived from the 15× sequencing data. As illustrated in Fig. 1c and detailed in Supplementary Table S5, MEHunter exhibited superior capability in identifying MEVs across all AF categories compared to rMETL and xTea. Notably, MEHunter identified over 33% more shared MEIs present on all haplotypes of the 32 individuals. Additionally, it identified over 12% more major, 22% more polymorphic, and 14% more singleton MEDs, where MEDs are defined as being present on at least half, two, or one of the haplotypes of the 32 individuals, respectively.

To rigorously evaluate the effectiveness of MEVs calling, we utilized a PacBio HiFi sequenced trio consisting of HG00731 (father), HG00732 (mother), and HG00733 (child) from the HGSVC2 project, analyzing the Mendelian Discordance Rate (MDR). The results presented in Fig. 1d and Supplementary Table S6, show that MEHunter exhibits significantly lower MDR values at 4.87% for MEVs (MEI: 5.68% and MED: 3.84%), compared to rMETL at 10.60% for MEVs (MEI: 14.10% and MED: 4.71%), and cuteSV at 9.06% for indels (insertion: 8.69% and deletion: 9.53%). This consistent superior performance underscores MEHunter’s robustness and accuracy in MEV detection under diverse experimental conditions.

Moreover, MEHunter also identified MEVs that were not supported by the ground truth data. As detailed in Supplementary Table S7, taking PacBio HiFi data as an example, approximately 6.3% (327/5209) of MEVs are categorized as false positives due to their inconsistent ME class with the ground truth, while another 14.0% (729/5209) are categorized as false positives due to complete discrepancies in the variant loci. Despite this, many of these unsupported calls still display strong evidence. For example, as shown in Supplementary Fig. S5, a 309 bp heterozygous Alu deletion was reported in 11 individuals, but not in HG00731. Nonetheless, multiple deletion signatures around 310 bp were clearly visible in the alignments for HG00731, and the corresponding consensus sequence closely matched an Alu element. This indicates that MEHunter can identify novel, potentially individual-specific MEVs that might have been overlooked in previous population studies.

We are also keenly aware that MEHunter sometimes fails to detect standard SVs present in the ground truth data due to alignment errors, leading to the misidentification of these variants as MEVs based on erroneous signatures (as detailed in Supplementary Fig. S6). It is worth noting that other alignment-based SV callers, such as Pacific Biosciences Structural Variant calling and analysis tools (PBSV) (https://github.com/PacificBiosciences/pbsv) and DeBreak (Chen et al. 2023), also fail to detect these SVs. To resolve this issue, a more robust strategy may be required, such as an assembly-based approach, which can meticulously parse subtle and error-prone SV signatures to accurately identify genuine MEVs. While offering greater precision, it is resource-intensive, requiring significant computational power and potentially additional sequencing efforts.

Finally, we assessed the elapsed time and memory footprints using 1, 2, 4, 8, and 16 Central Processing Unit (CPU) threads on the 15× HG00731 PacBio HiFi data as shown in Fig. 1e and Supplementary Tables S8 and S9. Overall, MEHunter demonstrated linear acceleration with increasing CPU threads and maintained relatively stable peak memory usage that was less than 32 GB. While the increased resource expenditure is primarily due to the intensive workload of deep learning-based inference, it remains within acceptable limits for execution on mainstream desktop computers.

In summary, MEHunter is a high-performance MEV detection approach that is enhanced by a fine-tuned transformer model along with several advanced algorithms. MEHunter excels in identifying potential MEVs, particularly those with fragmented features, and consistently achieves higher accuracy and sensitivity compared to state-of-the-art tools. This capability significantly improves the detection of potentially individual-specific MEVs that have been overlooked in previous population studies. However, MEHunter encounters challenges in accurately identifying the correct MEVs when faced with erroneous alignments. Addressing these challenges is a vital direction for future improvements to MEHunter. We anticipate that MEHunter will integrate seamlessly into current long-read sequencing data analysis pipelines, thereby advancing genetic research and technological development.

## Supplementary Material

btae557_Supplementary_Data

## Data Availability

The data used for benchmarking in this manuscript is available in our Supplementary Table S10.
