# Supplementary material for: MEHunter: transformer-based mobile element variant detection from long reads
Source: Bioinformatics. 2024 Sep 16;40(9):btae557. doi: 10.1093/bioinformatics/btae557 (PMC11415824; doi:10.1093/bioinformatics/btae557)
Supplement: btae557_Supplementary_Data [file btae557_supplementary_data.pdf]

MEHunter: transformer-based mobile element variant detection from long reads

Supplementary Material

Tao Jiang[1, 2, +], Zuji Zhou[1, +], Zhendong Zhang[1], Shuqi Cao[1], Yadong Wang[1, 2, *] and Yadong Liu[1, 2, *]

[1]Center for Bioinformatics, Faculty of Computing, Harbin Institute of Technology, Harbin, Heilongjiang 150001, China

[1]Zhengzhou Research Institute, Harbin Institute of Technology, Zhengzhou, Henan, 450000, China

*To whom correspondence should be addressed.

[+]These authors should be regarded as Joint First Authors.

Contents

Supplementary Fig. 1. A schematic illustration of MEHunter approach. 3
Supplementary Fig. 2. Schematic illustrations on the fine-tuned DNABERT2. 4
Supplementary Fig. 3. Venn diagrams of PacBio HiFi data- and ONT data-based callsets of HG00731. 5
Supplementary Fig. 4. A MEI example only correctly detected by MEHunter under its transformer model. 6
Supplementary Fig. 5. An example of an Alu deletion unreported in the ground truth for HG00731 but identified as an Alu by MEHunter in the same sample. 8
Supplementary Fig. 6. An example of a false positive call identified as a LINE1 by MEHunter. 9
Supplementary Table 1. Benchmark results on the simulated PacBio HiFi datasets. 10
Supplementary Table 2. Benchmark results on the simulated ONT datasets. 11
Supplementary Table 3. Benchmark results on the HG00731 PacBio HiFi datasets 12
Supplementary Table 4. Benchmark results on the HG00731 ONT datasets 13
Supplementary Table 5. The identification rate of each tool for 15× HG00731 data under different population-scale variant categories 14
Supplementary Table 6. The MDR benchmark results on a Trio data (HG00731, HG00732, HG00733) through PacBio HiFi sequencing. 15
Supplementary Table 7. Distribution of MEVs reported by MEHunter for the HG00731 sample, according to the consistency with established ground truth data. 16
Supplementary Table 8. The runtime benchmarking results on the 15× HG00731 datasets (minutes). 17
Supplementary Table 9. The memory footprints benchmarking results on the HG00731 datasets (GB). 18
Supplementary Table 10. Data availability. 19
Supplementary Notes 20

1. Supplementary Methods 20

1.1 Integrate cuteSV for calling SV 20

1.2 Calculate consensus with abPOA 20

1.3 Fast calculating identity score with a modified SW algorithm 20

1.4 Potential MEV prediction with minimap2 and fine-tuned DNABERT2 21

1.5 The implementation and fine-tuning of DNABERT2 21

2. Implementation of Benchmarking 22

2.1 Implementation of simulated data 22

2.2 Evaluation of MEV callsets 22

2.3 The command lines for benchmarking 23

References 27

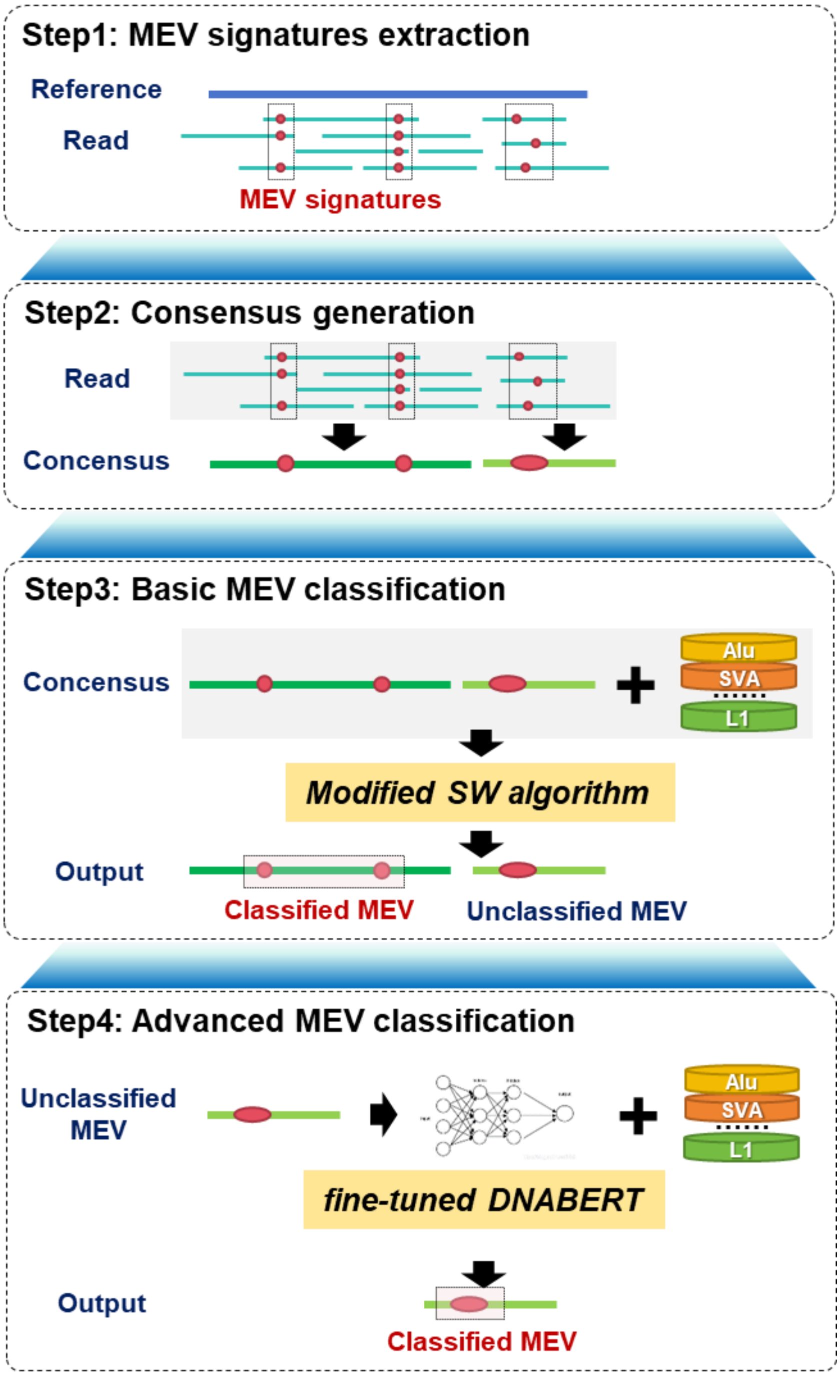

Supplementary Fig. 1. A schematic illustration of MEHunter approach.

MEHunter processes sorted BAM files to detect MEVs through four major steps. In the first step, "MEV Signatures Extraction," MEHunter utilizes cuteSV (Jiang, et al., 2020) to extract INDEL loci and signatures. The second step, "Consensus Generation," involves using abPOA (Gao, et al., 2021) to generate a consensus sequence for each cluster. The third step, "Basic MEV Classification," employs a lightweight Smith-Waterman (SW) algorithm designed by MEHunter to quickly output high-quality ME variants. In the final step, "Advanced MEV Classification," MEHunter uses minimap2 as a pre-classifier to exclude completely unrelated sequences and applies fine-tuned DNABERT2 (Zhou, et al., 2023) to enhance the detection of potential ME variants. This structured approach ensures efficient and accurate ME variant identification.

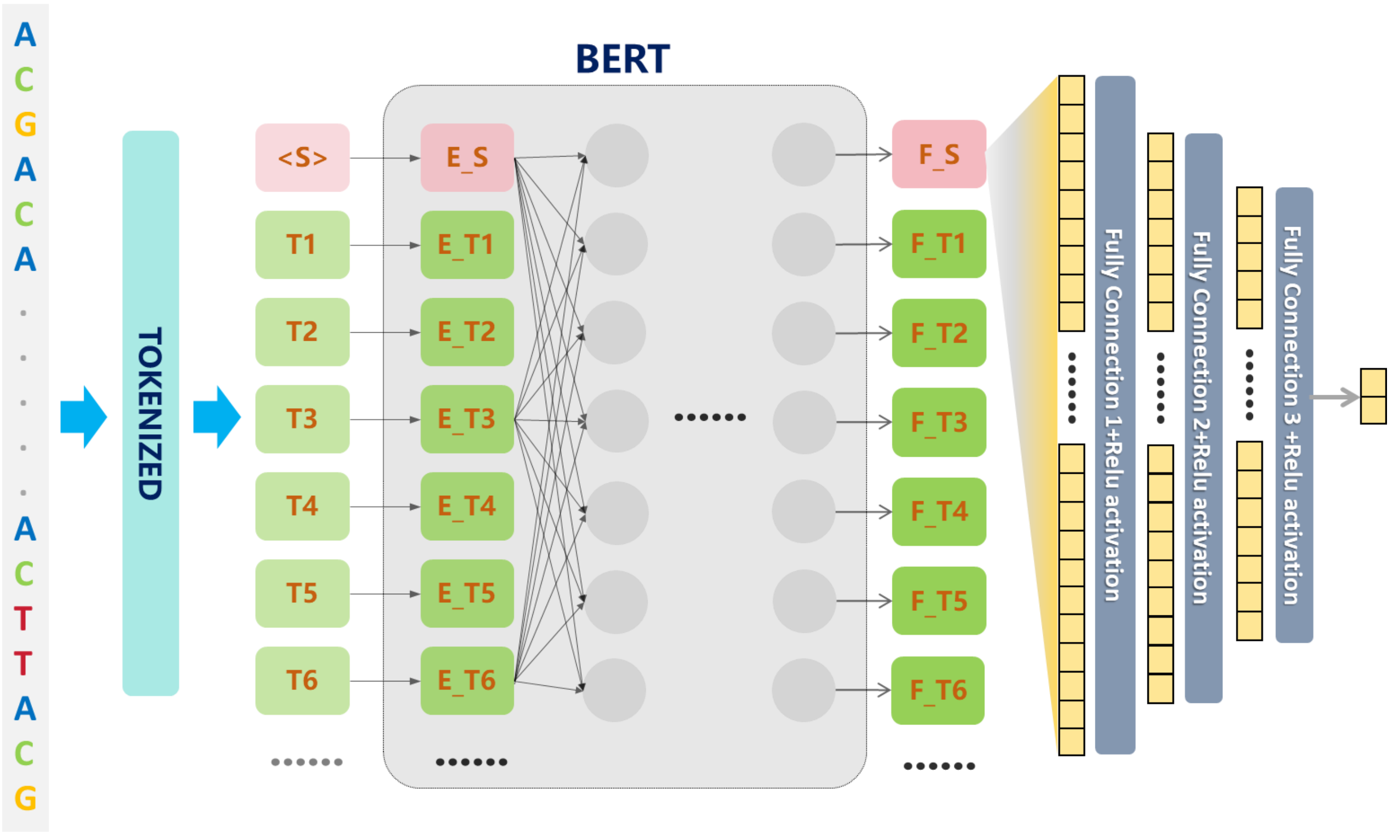

Supplementary Fig. 2. Schematic illustrations on the fine-tuned DNABERT2.

DNABERT2 processes base sequences by first encoding them into character sequences and subsequently converting these into word vectors. Specifically, the model takes the base sequence as input and translates it into a corresponding sequence of word vectors. The word vector linked to the 'cls' token is then fed into a fully connected network, which ultimately outputs a two-dimensional vector. This vector serves as the foundation for sequence classification, providing a structured and efficient approach to analyze genetic data.

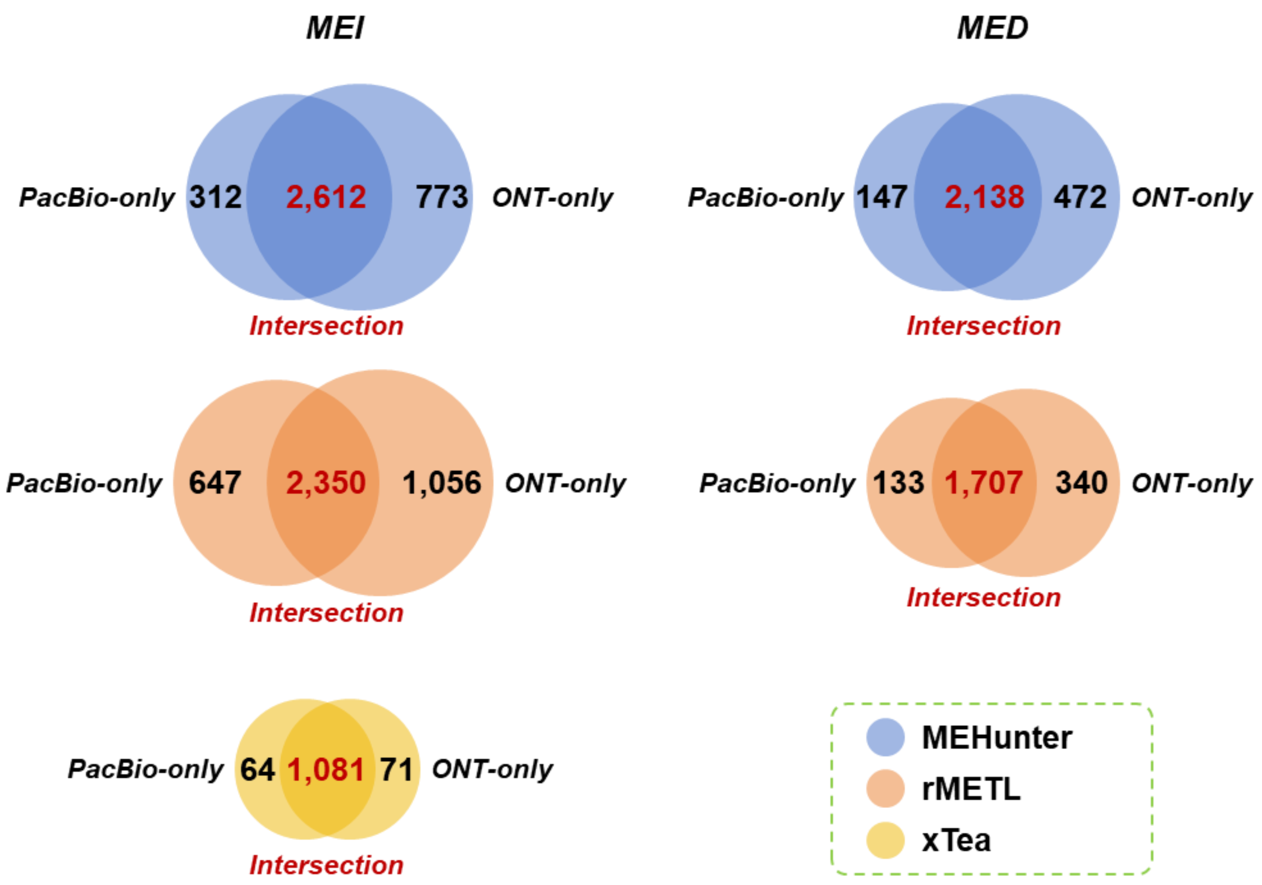

Supplementary Fig. 3. Venn diagrams of PacBio HiFi data- and ONT data-based callsets of HG00731.

The Venn diagrams on the left are the comparison results of MEI, and those of MED are on the right. The Blue, orange, and yellow represent MEHunter, rMETL, and xTea, respectively. PacBio-only and ONT-only are the MEI/MED that is only detected on the PacBio HiFi data and ONT data of the corresponding ME caller. Intersection indicates the MEI/MED that can be simultaneously identified on both sequencing data. The comparison of MED for xTea is not displayed since xTea cannot detect MED.

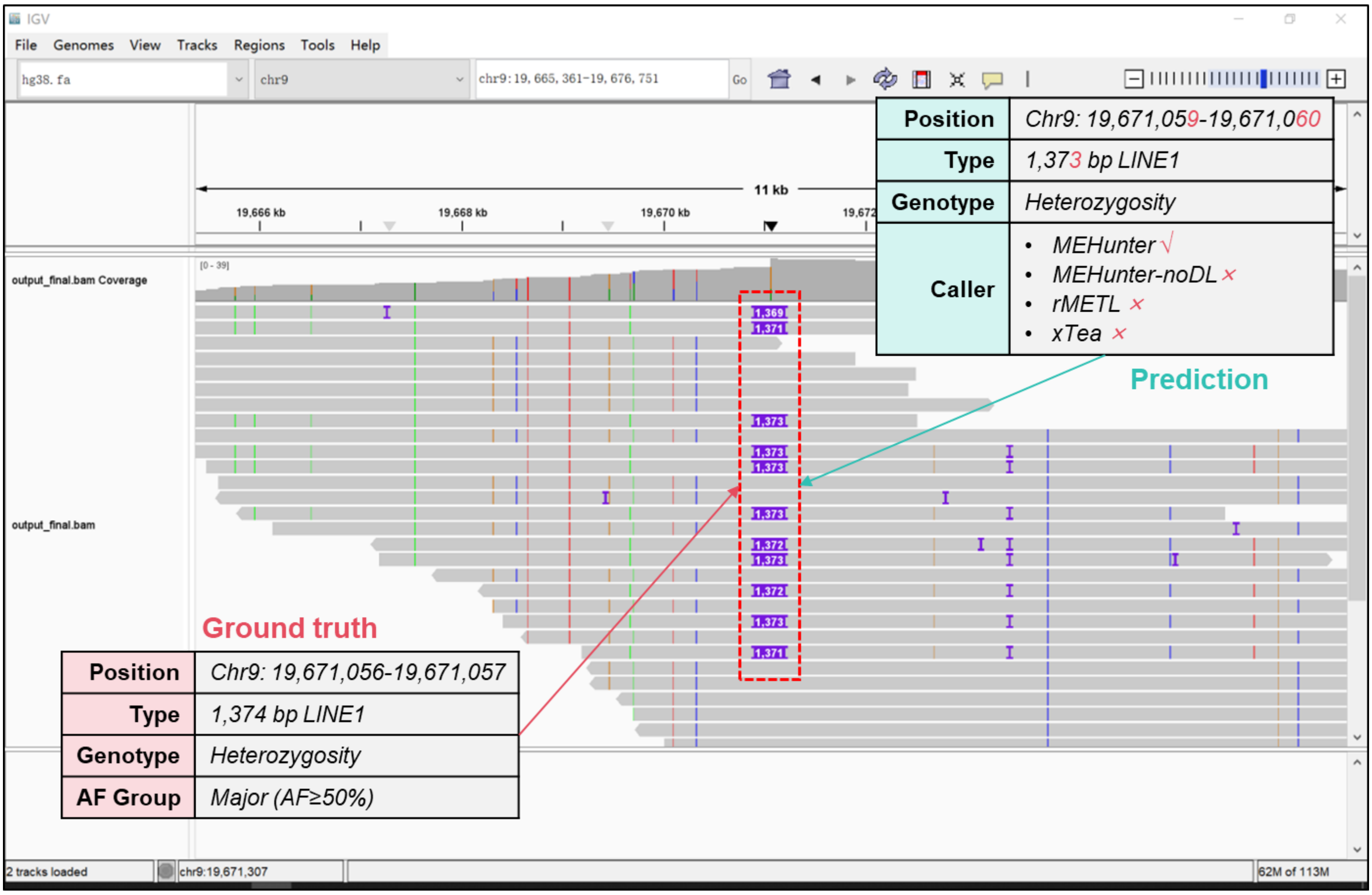

Supplementary Fig. 4. A MEI example only correctly detected by MEHunter under its transformer model.

The snapshot of Integrated Genomics Viewer (IGV) for the read alignments around a 1,374 bp heterozygous LINE 1 insertion (breakpoint at chr9: 19,671,056). This LINE 1 insertion event is only called by MEHunter under its minimap2-DNABERT2 module. This heterozygous LINE 1 insertion also presence on the haplotype of *HG00731, HG00732, HG00512, HG00513, NA12878, HG03486, HG03371, NA12329, HG00171, NA18939, HG03732, HG00096, NA20847, NA20509, HG00864, HG01505, NA18534, NA19650, HG01596, HG01114, HG02492*. The generated consensus of the inserted fragment is
"*ACAGTGCTCAATAATTATTTGTTGAATACATCAATAAGTAGGAATAGCATTTAAAAATTAGATGTATTAAAAAGCTTGGAAGGCTATA*
ACCTCTTACATTAACAACAGTCATCTCCGAGTGATAAAATAATAGGTAGTTTTTGTTTTCTTTTTTTATTTAAATAGGTTTTGGGGGG
TGCAAGTGGTGTTTTGTTACATAAATAAGTTCTTTACATGGGATTTCTGTGATTTTGGCACAGCCATCACCTGAGCAGTGTACAGT
GTACTCAATGTGTAGTCTTATAACCTTCATCCCCTCCTACACTTTCCCCCAAATTCCCAAAGTCCATTGTATCATTCTTATGCCTTT
GCATCCTCATAGCTTAGCTCCCACTTATGAGTGAGAACATATGATGTTTGGTTTTCCATTCCTCAGTAACTTCACTTAGAATAATAG
TCTTCAATCCCATCCAGGTTGCTGCGAATGCCATTAATTCATTCCTTTTTATGGCTGAAGTCCTCACAGTTTAGCTCCCAAAATTAA
TGGACACAAATCAGTAGTTCTGCTATACACCAACAGTGACCAAGCTGTGAATGAAATCAAGAACTCAACTCCTTTTACAATAGCTG
CAAAATAAATAAATAAATAAACAAACAAACCTTAGGAACATACCTAACCAAGGAGGCGAAAGACCTCTACAAGGAAAACTATGAAA
CACTGCTGAAGGAAATCATAGATGACACAAACAAATGGAAACACATCCCATGCTCATGGATGGGTAGAATCAATATTGTGAAAGT
GACCCTGTTGCCAAAAGCAATCTACAAATTCAACGCAATTTCCATCCAAATACCACCATCATTCTTCACATATGATGTTTAGTTTTC

CATTCCTGAGTTACTTCACTTAGAATAATAGTCTCCAATTCCATCCAGGTTGCTGCAAATGCCATTATTTCATTCCTTTTTATTGCTG

AGTAGTATTCCATGGTGCATGTGTGTGTGTGTGTGTGTGTGTGTGTGTGTGTATATATATAATCATATATATACGATATATGTGTACAT

ATATCATATATATATGTGTATATATATATATGTGTGTATATATATCATATATATGATACATATATTACAATTTCTTTATTCATTTGTTGATTGATGG

GCATTTGGGCTGGTTCCATATTTTTGCAATTGCAAATTGTGCTGCTATAAACACATATGTGAAAGTATCTCTCTTGTATGATGTCTT

CTTTTCCTCTGGGTAGATACCCAGGAGTGAGACTGCTGTATTAAATGGTAGTTCTACTTTTAGTTCTCTAAGAAATCTTCACACTG

TTTTCCATAGTGGTTGTACTAGTTTACATTTCCACTAGCAGTTTGAAAGAAGTAACTCAGGAA”.

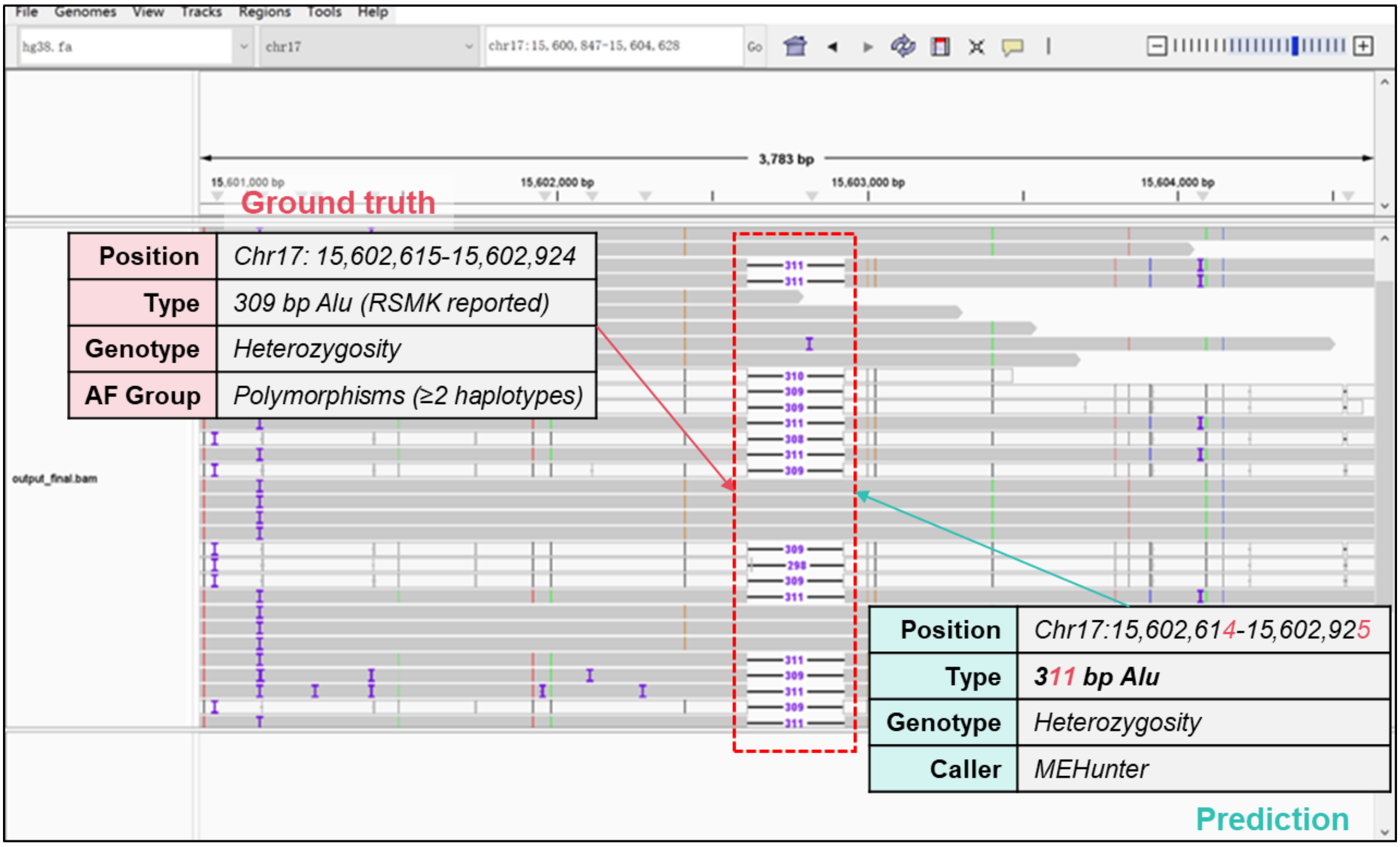

Supplementary Fig. 5. An example of an Alu deletion unreported in the ground truth for HG00731 but identified as an Alu by MEHunter in the same sample.

The snapshot of Integrated Genomics Viewer (IGV) for the read alignments around a 309 bp heterozygous deletion (breakpoint at chr17: 15,602,615). This deletion event is predicted as an Alu by MEHunter on the HG00731, whereas the ground truth only reported its presence on the haplotype of *HG00513, HG03125, HG03486, HG02818, HG03683, NA18939, HG03732, NA19650, HG02587, HG01596,* *NA19983*. The corresponding deleted fragment is "*TGAGACCGAGTTTTTTTTTTTTTTTTTTTTTTGAGACGGAGTCTCGCTGTCGCCCAGGCTGGAGTGCAGTGGCGCAATCTCGGC TCACTGCAGCCTCCGCCCCCTGGGGTTCACGCCATTCTCCTGCCTCAGCCTCCCGAGTAGCTGGGACTACAGGCGCCCGCCA CCTCGCCCGGCTAATTTTTTGTATTTTCAGTAGAGACGGGGTTTCACCGTGTTAGCCAGGATGGTCTCGATCTCCTGACCTCGT GATCCGCCCGCCTCGGCCTCCCAAAGTGCTGGGATTACAGGCGTGAGCCACCGCGCCCGGC*".

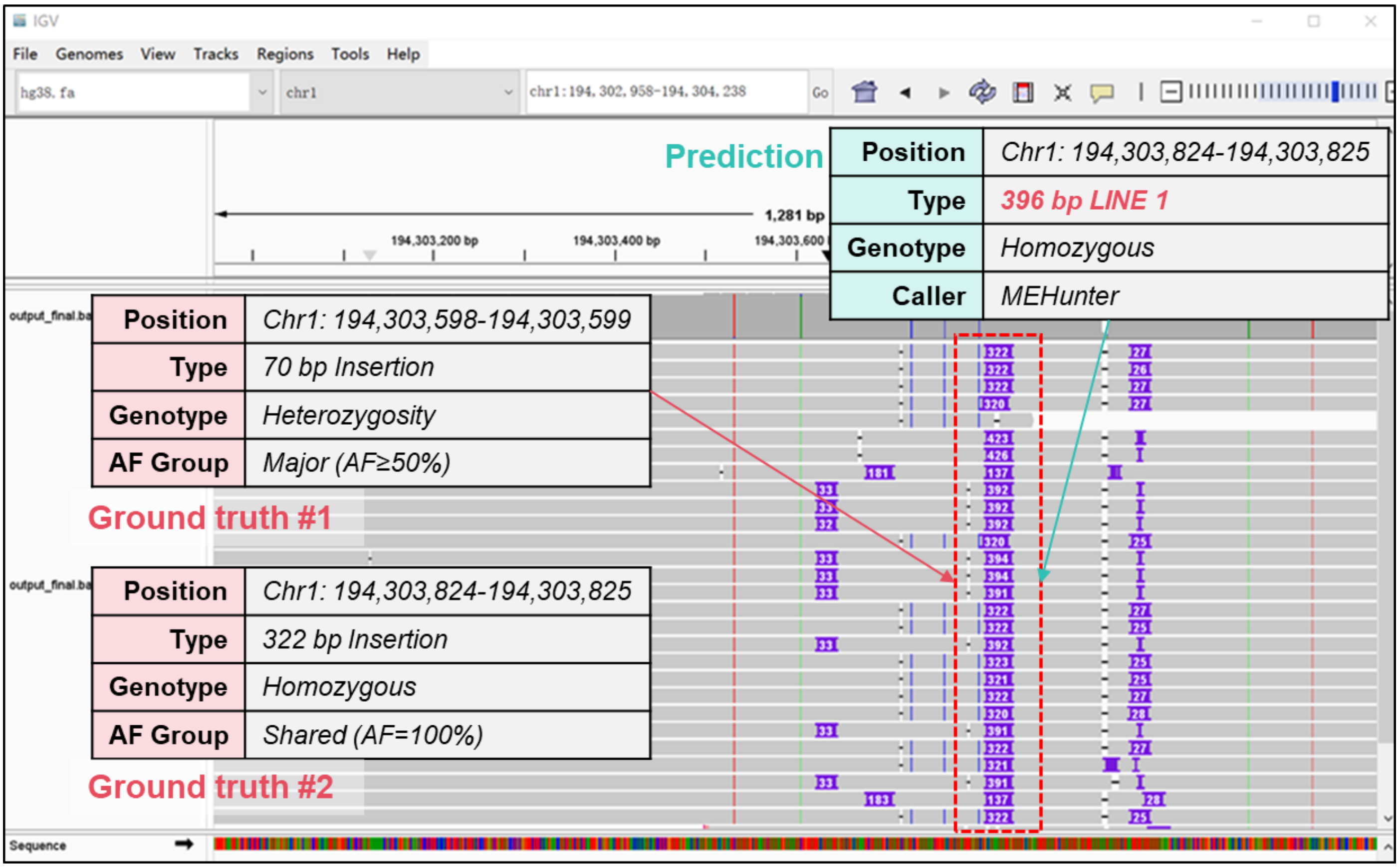

Supplementary Fig. 6. An example of a false positive call identified as a LINE1 by MEHunter.

The snapshot of Integrated Genomics Viewer (IGV) for the read alignments around two different insertions, one is a 70 bp heterozygous insertion (breakpoint at chr1: 194,303,598) and another one is a 322 bp homozygous insertion (breakpoint at chr1: 194,303,824). Although both two insertions are present on the HG00731 as reported by the ground truth established through diploid assembly, it is difficult for alignment-based SV caller to figure out this complex event, since the aligner treated them as a single SV (there are many about 390 bp insertion signatures shown in the IGV). Hence, MEHunter incorrectly predicted a 396 bp insertion and further determined it as a LINE 1. It is worth noting that other alignment-based SV callers like PBSV (https://github.com/PacificBiosciences/pbsv) and DeBreak (Chen, et al., 2023) also failed to detect them. The generated consensus of the fake inserted fragment is "*ATTCTTATGTATTATATATTTGTACATATATAAATATATTCTTATGTATTATATATTTGTACATATATAAATATATTCTTATGTATTATATATTTGTACATATATAAATATATTCTTATGTATTATATATTTGTACATATATAAATATATTCTTATGTATTATATATTTGTACATATATAAATATATTTTTATGTATTATATATTTGTACATATATAAATATATTTTTATGTATTATATATTTGTACATATATAAATATATTTTTATGTATTATATATTTGTACATATATAAATTTTTATGTATTATATATTTGTATATATA*".

Supplementary Table 1. Benchmark results on the simulated PacBio HiFi datasets.

		Precision	Recall	F1	FDR	GT-Precision	GT-Recall	GT-F1	GT-FDR
**Coverage = 5× (-s 1)**									
MEI	MEHunter	**99.98%**	**92.73%**	**96.22%**	**0.02%**	84.37%	**78.25%**	**81.19%**	15.63%
	rMETL	99.75%	56.49%	72.13%	0.25%	**91.32%**	51.72%	66.03%	**8.68%**
	xTea	96.41%	51.25%	66.76%	3.59%	N/A	N/A	N/A	N/A
	Palmer2	76.62%	68.93%	72.57%	23.38%	N/A	N/A	N/A	N/A
MED	MEHunter	**99.92%**	**95.24%**	**97.53%**	**0.08%**	**84.84%**	**80.87%**	**82.81%**	**15.16%**
	rMETL	99.76%	53.06%	69.28%	0.24%	66.18%	35.2%	45.96%	33.82%
**Coverage = 10× (-s 2)**									
MEI	MEHunter	**99.99%**	**95.72%**	**97.81%**	**0.01%**	92.92%	**88.95%**	**90.89%**	7.08%
	rMETL	99.45%	61.69%	76.15%	0.55%	**94.91%**	58.87%	72.67%	**5.09%**
	xTea	97.51%	58.44%	73.08%	2.49%	N/A	N/A	N/A	N/A
	Palmer2	79.83%	74.12%	76.87%	20.17%	N/A	N/A	N/A	N/A
MED	MEHunter	**99.92%**	**97.58%**	**98.73%**	**0.08%**	**93.23%**	**91.04%**	**92.12%**	**6.77%**
	rMETL	99.82%	54.00%	70.26%	0.18%	63.66%	34.58%	44.81%	36.34%
**Coverage = 20× (-s 3)**									
MEI	MEHunter	**99.98%**	**98.53%**	**99.25%**	**0.02%**	97.29%	**95.87%**	**96.57%**	2.71%
	rMETL	99.25%	65.37%	78.82%	0.75%	**97.39%**	64.14%	77.34%	**2.61%**
	xTea	97.28%	61.75%	75.55%	2.72%	N/A	N/A	N/A	N/A
	Palmer2	82.41%	81.65%	82.03%	17.59%	N/A	N/A	N/A	N/A
MED	MEHunter	**99.92%**	**99.64%**	**99.78%**	**0.08%**	**97.66%**	**97.38%**	**97.52%**	**2.34%**
	rMETL	99.83%	55.49%	71.33%	0.17%	62.15%	34.55%	44.41%	37.85%
**Coverage = 30× (-s 5)**									
MEI	MEHunter	**99.99%**	**99.00%**	**99.42%**	**0.01%**	**99.00%**	**97.87%**	**98.43%**	**1.00%**
	rMETL	99.06%	66.94%	79.89%	0.94%	97.68%	66.01%	78.78%	2.32%
	xTea	96.93%	68.5%	80.27%	3.07%	N/A	N/A	N/A	N/A
	Palmer2	81.97%	83.94%	82.94%	18.03%	N/A	N/A	N/A	N/A
MED	MEHunter	**99.94%**	**99.74%**	**99.84%**	**0.06%**	**99.27%**	**99.08%**	**99.17%**	**0.73%**
	rMETL	99.88%	55.53%	71.38%	0.12%	61.15%	34.00%	43.70%	38.85%

The best results have been marked in bold. The evaluation results were computed by our in-house scripts which are available at https://github.com/120L021101/MEHunter_experiment

Supplementary Table 2. Benchmark results on the simulated ONT datasets.

		Precision	Recall	F1	FDR	GT-Precision	GT-Recall	GT-F1	GT-FDR
**Coverage = 5× (-s 1)**									
MEI	MEHunter	**99.98%**	**94.58%**	**97.20%**	**0.02%**	84.45%	**79.89%**	**82.11%**	15.55%
	rMETL	99.56%	53.42%	69.53%	0.44%	**91.48%**	49.09%	63.89%	**8.52%**
	xTea	97.08%	32.1%	48.25%	2.92%	N/A	N/A	N/A	N/A
	Palmer2	75.33%	52.18%	61.74%	24.67%	N/A	N/A	N/A	N/A
MED	MEHunter	**99.60%**	**95.23%**	**97.37%**	**0.40%**	**84.97%**	**81.24%**	**83.06%**	**15.03%**
	rMETL	93.97%	53.02%	67.79%	6.03%	82.95%	46.80%	59.84%	17.05%
**Coverage = 10× (-s 2)**									
MEI	MEHunter	**99.99%**	**96.72%**	**98.33%**	**0.01%**	93.37%	**90.31%**	**91.82%**	6.63%
	rMETL	99.39%	56.83%	72.31%	0.61%	**96.18%**	55.00%	69.98%	**3.82%**
	xTea	98.12%	41.01%	57.87%	1.88%	N/A	N/A	N/A	N/A
	Palmer2	83.29%	61.47%	70.74%	16.71%	N/A	N/A	N/A	N/A
MED	MEHunter	**99.88%**	**97.87%**	**98.86%**	**0.12%**	**93.69%**	**91.80%**	**92.73%**	**6.31%**
	rMETL	96.70%	54.34%	69.58%	3.30%	89.55%	50.33%	64.44%	10.45%
**Coverage = 20× (-s 3)**									
MEI	MEHunter	**99.99%**	**99.10%**	**99.54%**	**0.01%**	97.60%	**96.73%**	**97.16%**	2.40%
	rMETL	98.94%	60.25%	74.89%	1.06%	**97.90%**	59.61%	74.10%	**2.10%**
	xTea	97.54%	48.62%	64.89%	2.46%	N/A	N/A	N/A	N/A
	Palmer2	86.02%	65.7%	74.5%	13.98%	N/A	N/A	N/A	N/A
MED	MEHunter	**99.91%**	**99.78%**	**99.84%**	**0.09%**	**97.78%**	**97.66%**	**97.72%**	**2.22%**
	rMETL	96.69%	55.43%	70.46%	3.31%	91.38%	52.39%	66.60%	8.62%
**Coverage = 30× (-s 5)**									
MEI	MEHunter	**99.99%**	**99.25%**	**99.62%**	**0.01%**	**99.20%**	**98.47%**	**98.83%**	**0.80%**
	rMETL	98.76%	61.66%	75.92%	1.24%	98.10%	61.25%	75.41%	1.90%
	xTea	98.23%	53.7%	69.44%	1.77%	N/A	N/A	N/A	N/A
	Palmer2	85.07%	67.39%	75.2%	14.93%	N/A	N/A	N/A	N/A
MED	MEHunter	**99.92%**	**99.88%**	**99.90%**	**0.08%**	**99.32%**	**99.29%**	**99.31%**	**0.68%**
	rMETL	97.41%	55.48%	70.70%	2.59%	92.27%	52.56%	66.97%	7.73%

The best results have been marked in bold. The evaluation results were computed by our in-house scripts which are available at https://github.com/120L021101/MEHunter_experiment

Supplementary Table 3. Benchmark results on the HG00731 PacBio HiFi datasets

		Precision	Recall	F1	FDR	GT-Precision	GT-Recall	GT-F1	GT-FDR
**Coverage = 5× (-s 1)**									
MEI	MEHunter	79.93%	**67.78%**	**73.35%**	20.07%	**67.16%**	**56.96%**	**61.64%**	**32.84%**
	MEHunter-nDL	70.1%	20.41%	31.61%	29.90%	57.78%	16.82%	26.06%	42.22%
	rMETL	77.08%	57.15%	65.64%	22.92%	63.50%	47.08%	54.07%	36.50%
	xTea	**92.45%**	15.63%	26.7%	**7.55%**	N/A	N/A	N/A	N/A
MED	MEHunter	85.19%	**69.66%**	**76.65%**	14.81%	74.50%	**60.92%**	**67.03%**	25.50%
	MEHunter-nDL	**85.71%**	24.88%	38.57%	**14.29%**	**76.65%**	22.25%	34.5%	**23.35%**
	rMETL	82.41%	56.59%	67.10%	17.59%	70.22%	48.22%	57.18%	29.78%
**Coverage = 10× (-s 2)**									
MEI	MEHunter	82.29%	**71.20%**	**76.35%**	17.71%	**74.18%**	**64.19%**	**68.83%**	**25.82%**
	MEHunter-nDL	71.63%	21.48%	33.05%	28.37%	64.78%	19.43%	29.89%	35.22%
	rMETL	76.67%	62.27%	68.72%	23.33%	66.88%	54.32%	59.94%	33.12%
	xTea	**93.12%**	20.76%	33.96%	**6.88%**	N/A	N/A	N/A	N/A
MED	MEHunter	86.83%	**71.55%**	**78.45%**	13.17%	80.31%	**66.18%**	**72.56%**	19.69%
	MEHunter-nDL	**87.26%**	25.62%	39.62%	**12.74%**	**81.96%**	24.07%	37.21%	**18.04%**
	rMETL	85.03%	57.22%	68.40%	14.97%	77.38%	52.07%	62.25%	22.62%
**Coverage = 15× (-s 3)**									
MEI	MEHunter	82.76%	**72.31%**	**77.18%**	17.24%	**77.08%**	**67.36%**	**71.89%**	**22.92%**
	MEHunter-nDL	72.95%	22.07%	33.89%	27.05%	68.21%	20.63%	31.68%	31.79%
	rMETL	77.92%	60.77%	68.29%	22.08%	69.39%	54.12%	60.81%	30.61%
	xTea	**93.5%**	30.48%	45.97%	**6.50%**	N/A	N/A	N/A	N/A
MED	MEHunter	87.92%	**71.70%**	**78.98%**	12.08%	83.56%	**68.14%**	**75.07%**	16.44%
	MEHunter-nDL	**88.98%**	26.03%	40.28%	**11.02%**	**86.07%**	25.18%	38.96%	**13.93%**
	rMETL	88.00%	55.18%	67.83%	12.00%	81.68%	51.22%	62.96%	18.32%
**Coverage = 36× (-s 5)**									
MEI	MEHunter	83.01%	**74.89%**	**78.74%**	16.99%	**80.44%**	**72.57%**	**76.31%**	**19.56%**
	MEHunter-nDL	74.0%	22.92%	35.0%	26.00%	72.94%	22.59%	34.5%	27.06%
	rMETL	73.17%	71.50%	72.32%	26.83%	65.09%	63.61%	64.34%	34.91%
	xTea	**93.88%**	35.05%	51.04%	**6.12%**	N/A	N/A	N/A	N/A
MED	MEHunter	88.37%	**73.51%**	**80.26%**	11.63%	86.86%	**72.25%**	**78.89%**	13.14%
	MEHunter-nDL	**89.75%**	26.29%	40.67%	**10.25%**	**88.49%**	25.92%	40.1%	**11.51%**
	rMETL	86.52%	58.96%	70.13%	13.48%	80.97%	55.18%	65.63%	19.03%

The best results have been marked in bold. MEHunter-nDL means performing MEHunter does not enable the deep learning module. The evaluation results were computed by our in-house scripts which are available at https://github.com/120L021101/MEHunter_experiment

Supplementary Table 4. Benchmark results on the HG00731 ONT datasets

		Precision	Recall	F1	FDR	GT-Precision	GT-Recall	GT-F1	GT-FDR
**Coverage = 5× (-s 1)**									
MEI	MEHunter	76.75%	**60.61%**	**67.73%**	23.25%	**66.18%**	**52.26%**	**58.40%**	**33.82%**
	MEHunter-nDL	68.91%	18.29%	28.91%	31.09%	57.12%	15.16%	23.96%	42.88%
	rMETL	74.97%	47.27%	57.98%	25.03%	62.20%	39.22%	48.11%	37.80%
	xTea	**90.8%**	17.53%	29.39%	**9.20%**	N/A	N/A	N/A	N/A
MED	MEHunter	81.70%	**60.88%**	**69.77%**	18.30%	69.33%	**51.66%**	**59.21%**	30.67%
	MEHunter-nDL	**83.68%**	21.85%	34.65%	**16.32%**	**74.46%**	19.44%	30.83%	**25.54%**
	rMETL	82.38%	49.18%	61.59%	17.62%	72.39%	43.22%	54.12%	27.61%
**Coverage = 10× (-s 2)**									
MEI	MEHunter	76.85%	**70.36%**	**73.46%**	23.15%	**70.15%**	**64.23%**	**67.06%**	**29.85%**
	MEHunter-nDL	67.46%	21.22%	32.29%	32.54%	62.59%	19.69%	29.96%	37.41%
	rMETL	74.76%	57.67%	65.12%	25.24%	64.70%	49.91%	56.35%	35.30%
	xTea	**91.47%**	20.33%	33.27%	**8.53%**	N/A	N/A	N/A	N/A
MED	MEHunter	82.48%	**68.88%**	**75.07%**	17.52%	75.65%	**63.18%**	**68.85%**	24.35%
	MEHunter-nDL	**84.26%**	24.59%	38.07%	**15.74%**	**80.32%**	23.44%	36.29%	**19.68%**
	rMETL	82.87%	55.03%	66.14%	17.13%	75.62%	50.22%	60.36%	24.38%
**Coverage = 15× (-s 3)**									
MEI	MEHunter	75.47%	**76.98%**	**76.22%**	24.53%	**71.25%**	**72.67%**	**71.96%**	**28.75%**
	MEHunter-nDL	67.51%	23.44%	34.8%	32.49%	64.6%	22.43%	33.3%	35.40%
	rMETL	70.46%	66.67%	68.52%	29.54%	61.30%	58.00%	59.60%	38.70%
	xTea	**91.62%**	27.84%	42.7%	**8.38%**	N/A	N/A	N/A	N/A
MED	MEHunter	80.36%	**74.88%**	**77.53%**	19.64%	75.87%	**70.70%**	**73.19%**	24.13%
	MEHunter-nDL	**82.14%**	26.4%	39.96%	**17.86%**	**79.14%**	25.44%	38.5%	**20.86%**
	rMETL	79.55%	60.11%	68.48%	20.45%	73.48%	55.51%	63.24%	26.52%
**Coverage = 39× (-s 7)**									
MEI	MEHunter	76.14%	**77.76%**	**76.94%**	23.86%	**74.20%**	**75.77%**	**74.97%**	**25.80%**
	MEHunter-nDL	68.57%	23.83%	35.37%	31.43%	67.82%	23.57%	34.98%	32.18%
	rMETL	64.97%	72.15%	68.37%	35.03%	57.51%	63.87%	60.52%	42.49%
	xTea	**92.15%**	34.59%	50.3%	**7.85%**	N/A	N/A	N/A	N/A
MED	MEHunter	80.38%	**75.59%**	**77.91%**	19.62%	77.94%	**73.29%**	**75.54%**	22.06%
	MEHunter-nDL	**83.39%**	26.77%	40.53%	**16.61%**	**82.0%**	26.33%	39.86%	**18.00%**
	rMETL	79.87%	60.55%	68.88%	20.13%	74.93%	56.81%	64.63%	25.07%

The best results have been marked in bold. MEHunter-nDL means performing MEHunter does not enable the deep learning module. The evaluation results were computed by our in-house scripts which are available at https://github.com/120L021101/MEHunter_experiment

Supplementary Table 5. The identification rate of each tool for 15× HG00731 data under different population-scale variant categories

		PacBio HiFi			ONT		
		MEHunter	rMETL	xTea	MEHunter	rMETL	xTea
**Shared (AF=100%)**	MEI	**75.17%**	41.43%	10.83%	**80.06%**	42.83%	12.52%
	MED	**87.73%**	78.74%	N/A	**91.28%**	81.47%	N/A
**Major (AF≥50%)**	MEI	**69.79%**	62.50%	31.83%	**75.06%**	70.18%	26.69%
	MED	**74.65%**	60.58%	N/A	**77.75%**	65.39%	N/A
**Polymorphisms (≥2 haplotypes)**	MEI	**76.15%**	70.96%	38.98%	**79.88%**	77.96%	30.04%
	MED	**59.53%**	33.23%	N/A	**62.71%**	39.88%	N/A
**Singletons (unique)**	MEI	**83.78%**	82.43%	52.7%	**83.78%**	79.72%	54.27%
	MED	**44.44%**	30.15%	N/A	**46.03%**	28.57%	N/A

The best results have been marked in bold. The evaluation results were computed by our in-house scripts which are available at https://github.com/120L021101/MEHunter_experiment

Supplementary Table 6. The MDR benchmark results on a Trio data (HG00731, HG00732, HG00733) through PacBio HiFi sequencing.

	Consistent MEI/INS	Consistent MED/DEL	Inconsistent MEI/INS	Inconsistent MED/DEL	MEI/INS MDR	MED/DEL MDR	Total MDR
MEHunter	2,270	2,252	167	90	**5.68%**	**3.84%**	**4.87%**
rMETL	2,767	1,820	454	90	14.10%	4.71%	10.60%
cuteSV	18,817	14,461	1,790	1,524	8.69%	9.53%	9.06%

The best results have been marked in bold. The evaluation results were computed by our in-house scripts which are available at https://github.com/120L021101/MEHunter_experiment

Supplementary Table 7. Distribution of MEVs reported by MEHunter for the HG00731 sample, according to the consistency with established ground truth data.

	PacBio HiFi			ONT		
	**MEI**	**MED**	**Total**	**MEI**	**MED**	**Total**
**TP call & TP class**	2,217	1,936	4,153	2,361	2,022	4,383
**TP call & FP class**	244	83	327	257	94	351
**FP**	463	266	729	767	494	1,261
**Total**	2,924	2,285	5,209	3,385	2,610	5,995

TP call indicates the variant locus for the corresponding MEV can be correctly detected by MEHunter. TP class and FP class indicate the ME type for the corresponding MEV can be correctly or incorrectly detected by MEHunter. The evaluation results were computed by our in-house scripts which are available at https://github.com/120L021101/MEHunter_experiment

Supplementary Table 8. The runtime benchmarking results on the 15× HG00731 datasets (minutes).

Platform	Threading Number	1	2	4	8	16
PacBio HiFi	MEHunter	143	80	51	35	26
	rMETL	**21**	**12**	**10**	**9**	**10**
	xTea	>1440 (24h)	>1440 (24h)	986	602	473
ONT	MEHunter	133	85	53	34	21
	rMETL	**41**	**33**	**25**	**24**	**18**
	xTea	>1440 (24h)	>1440 (24h)	1031	787	519

The best results have been marked in bold. The evaluation results were computed by our in-house scripts which are available at https://github.com/120L021101/MEHunter_experiment

Supplementary Table 9. The memory footprints benchmarking results on the HG00731 datasets (GB).

Platform	Threading Number	1	2	4	8	16
PacBio HiFi	MEHunter	21.97	22.35	23.97	26.06	28.19
	rMETL	**3.37**	**3.44**	**3.58**	**3.87**	**5.89**
	xTea	N/A	N/A	9.08	16.82	22.75
ONT	MEHunter	24.13	26.84	27.93	30.76	31.77
	rMETL	**5.50**	**5.75**	**6.20**	**7.12**	**8.99**
	xTea	N/A	N/A	10.94	18.15	24.33

The best results have been marked in bold. The evaluation results were computed by our in-house scripts which are available at https://github.com/120L021101/MEHunter_experiment.

Supplementary Table 10. Data availability.

Dataset	Link
hs37d5.fa.gz	https://ftp.1000genomes.ebi.ac.uk/vol1/ftp/technical/reference/phase2_reference_assembly_sequence/hs37d5.fa.gz
HG00731 PacBio HiFi data	http://ftp.1000genomes.ebi.ac.uk/vol1/ftp/data_collections/HGSVC2/working/20190925_PUR_PacBio_HiFi/HG00731_20190925_EEE_m54329U_190528_231241.Q20.fastq.gz<br>http://ftp.1000genomes.ebi.ac.uk/vol1/ftp/data_collections/HGSVC2/working/20190925_PUR_PacBio_HiFi/HG00731_20190925_EEE_m54329U_190531_175004.Q20.fastq.gz<br>http://ftp.1000genomes.ebi.ac.uk/vol1/ftp/data_collections/HGSVC2/working/20190925_PUR_PacBio_HiFi/HG00731_20190925_EEE_m54329U_190601_235636.Q20.fastq.gz<br>http://ftp.1000genomes.ebi.ac.uk/vol1/ftp/data_collections/HGSVC2/working/20190925_PUR_PacBio_HiFi/HG00731_20190925_EEE_m54329U_190606_045526.Q20.fastq.gz<br>http://ftp.1000genomes.ebi.ac.uk/vol1/ftp/data_collections/HGSVC2/working/20190925_PUR_PacBio_HiFi/HG00731_20190925_EEE_m54329U_190906_205127.Q20.fastq.gz
HG00731 PacBio ONT data	http://ftp.1000genomes.ebi.ac.uk/vol1/ftp/data_collections/HGSVC3/working/20211013_ONT_Rebasecalled/HG00731/20220510_220504_21-lee-006_PC24B149_1D_guppy-5.0.11-sup-prom_fastq_pass.fastq.gz<br>http://ftp.1000genomes.ebi.ac.uk/vol1/ftp/data_collections/HGSVC3/working/20211013_ONT_Rebasecalled/HG00731/20220510_220504_21-lee-006_PC24B149_3C_guppy-5.0.11-sup-prom_fastq_pass.fastq.gz<br>http://ftp.1000genomes.ebi.ac.uk/vol1/ftp/data_collections/HGSVC3/working/20211013_ONT_Rebasecalled/HG00731/20220510_220505_21-lee-006_PC24B149_3C_guppy-5.0.11-sup-prom_fastq_pass.fastq.gz<br>http://ftp.1000genomes.ebi.ac.uk/vol1/ftp/data_collections/HGSVC3/working/20211013_ONT_Rebasecalled/HG00731/20220511_220506_21-lee-006_PC24B149_1D_guppy-5.0.11-sup-prom_fastq_pass.fastq.gz
HG00732 PacBio HiFi data	http://ftp.1000genomes.ebi.ac.uk/vol1/ftp/data_collections/HGSVC2/working/20190925_PUR_PacBio_HiFi/HG00732_20190925_EEE_m54329U_190604_224858.Q20.fastq.gz<br>http://ftp.1000genomes.ebi.ac.uk/vol1/ftp/data_collections/HGSVC2/working/20190925_PUR_PacBio_HiFi/HG00732_20190925_EEE_m54329U_190610_071123.Q20.fastq.gz<br>http://ftp.1000genomes.ebi.ac.uk/vol1/ftp/data_collections/HGSVC2/working/20190925_PUR_PacBio_HiFi/HG00732_20190925_EEE_m54329U_190611_132246.Q20.fastq.gz<br>http://ftp.1000genomes.ebi.ac.uk/vol1/ftp/data_collections/HGSVC2/working/20190925_PUR_PacBio_HiFi/HG00732_20190925_EEE_m54329U_190612_193411.Q20.fastq.gz<br>http://ftp.1000genomes.ebi.ac.uk/vol1/ftp/data_collections/HGSVC2/working/20190925_PUR_PacBio_HiFi/HG00732_20190925_EEE_m54329U_190705_000551.Q20.fastq.gz
HG00733 PacBio HiFi data	http://ftp.1000genomes.ebi.ac.uk/vol1/ftp/data_collections/HGSVC2/working/20190925_PUR_PacBio_HiFi/HG00733_20190925_EEE_m54329U_190607_185248.Q20.fastq.gz<br>http://ftp.1000genomes.ebi.ac.uk/vol1/ftp/data_collections/HGSVC2/working/20190925_PUR_PacBio_HiFi/HG00733_20190925_EEE_m54329U_190615_010947.Q20.fastq.gz<br>http://ftp.1000genomes.ebi.ac.uk/vol1/ftp/data_collections/HGSVC2/working/20190925_PUR_PacBio_HiFi/HG00733_20190925_EEE_m54329U_190617_231905.Q20.fastq.gz<br>http://ftp.1000genomes.ebi.ac.uk/vol1/ftp/data_collections/HGSVC2/working/20190925_PUR_PacBio_HiFi/HG00733_20190925_EEE_m54329U_190619_052546.Q20.fastq.gz<br>http://ftp.1000genomes.ebi.ac.uk/vol1/ftp/data_collections/HGSVC2/working/20190925_PUR_PacBio_HiFi/HG00733_20190925_EEE_m54329U_190629_180018.Q20.fastq.gz<br>http://ftp.1000genomes.ebi.ac.uk/vol1/ftp/data_collections/HGSVC2/working/20190925_PUR_PacBio_HiFi/HG00733_20190925_EEE_m54329U_190701_222759.Q20.fastq.gz<br>http://ftp.1000genomes.ebi.ac.uk/vol1/ftp/data_collections/HGSVC2/working/20190925_PUR_PacBio_HiFi/HG00733_20190925_EEE_m54329U_190827_173812.Q20.fastq.gz

Supplementary Notes

1. Supplementary Methods

1.1 Integrate cuteSV for calling SV

As cuteSV (Jiang, et al., 2020) is state-of-the-art in calling generic SVs, we integrate it to provide variant signals for succeeding modules. More specifically, we set parameters of cuteSV, *--diff_ratio_merging_INS* (do not merge breakpoints with base pair identity more than the ratio of default for insertion) *--diff_ratio_merging_DEL* (do not merge breakpoints with base pair identity more than the ratio of default for deletion) and *--diff_ratio_filtering_TRA* (filter breakpoints with base pair identity less than the ratio of default for translocation) to 1.1 in order to avoid calling translocations and fully output insertions and deletions (detailed descriptions of these parameters are available at https://github.com/tjiangHIT/cuteSV). CuteSV then provides variant information in vcf format outputs and further details of signatures in .sigs files.

1.2 Calculate consensus with abPOA

As randomly picking a read or sequence from clusters highly possibly leads to being affected by noisy sequencing errors and chimeric alignments, we calculated the consensus of each cluster or part cluster using abPOA (Gao, et al., 2021, detailed descriptions of the parameters are available at https://github.com/yangao07/abPOA). As the memory cost of multiple sequence alignment (MSA) algorithms is not linear relative to the number of sequences used, we have specifically designed a strategy to efficiently handle overly large clusters containing a substantial amount of sequences. The criteria for defining what constitutes a 'large cluster' and the methodology for optimizing these clusters are detailed below:

$$f(C) = \begin{cases} C, \ |C| \le L \\ \text{argmin}(F(S,C)), S \subseteq C, |S| = L, |C| > L \end{cases} \tag{1}$$

where L is a hyperparameter (default: 10), C refers to *Cluster* and $F(S,C)$ is

$$F(S,C) = \sum_{x_i \in S} \frac{1}{|S|}(x_i - \bar{y})^2, \quad \bar{y} = \frac{1}{|C|}\sum_{y_i \in C} y_i \tag{2}$$

1.3 Fast calculating identity score with a modified SW algorithm

Following Smith-Waterman algorithm (Ligowski and Rudnicki, 2009) of using dynamic programming to solve sequence alignment problems, we have designed a lightweight algorithm based on linear space complexity called MEHunter-Aligner to better adapt to the unique nature of the problem. Firstly, our problem does not require detailed sequence alignment, but only requires obtaining the similarity between two sequences, so we do not need an O(NM) array to record the optimal alignment, and can involve approximation algorithms within the tolerable range of accuracy loss. Secondly, based on the fact that in our problem scenario, the results of similarity calculation with known ME sequences are mostly only very similar and completely unrelated. Ambiguous situations can be handed over to downstream modules for further exploration. Therefore, a more relaxed loss function can be designed to accelerate the algorithm's running speed.

Therefore, our first change is to define a completely new loss function which is

$$Loss(S_1, S_2) = \frac{2 * max(INS, DEL)}{max(length(S_1), length(S_2))} \tag{3}$$

where S1, S2 represent two sequence inputs, INS and DEL represent the operations of converting one sequence into another, as defined in Smith Watson. Our second modification is to introduce an early stop strategy based on maximum loss, based on the fact that if two sequences are already sufficiently dissimilar, there is no need to continue running and waste computing resources. Therefore, the output of our algorithm is

$$MEHunterAligner(S_1, S_2) = \begin{cases} 1 - Loss(S_1, S_2), & Loss(S_1, S_2) < Th \\ 0, & Loss(S_1, S_2) \geq Th \end{cases} \quad (4)$$

where Th is the maximum loss threshold, a hyperparameter with a default value of 0.15.

1.4 Potential MEV prediction with minimap2 and fine-tuned DNABERT2

For the fact that the variant sequence is able to be aligned to known ME sequences if it is a real MEV, MEHunter integrates minimap2 (Li, 2018) for re-alignment. However, the opposite direction, only if, is not always true. As ME accounts for approximately 47% human genomes, it can't be neglected that it is possible for a large variant to carry some ME components, thus leading to a successful alignment. So, completely and only relying on minimap2 is not always reliable. Therefore, we fine-tuned DNABERT2 (Zhou, et al., 2023), a model that is able to study in-depth features under different circumstances and nuances between similar events and classify successfully re-aligned variant sequences to do a final filtration.

1.5 The implementation and fine-tuning of DNABERT2

We added a three-layer forward propagation network downstream of DNABERT2 and used RELU as the activation function, ultimately outputting a two-dimensional vector for final classification based on positive and negative results.

For fine-tuning DNABERT2 to better adapt to our task, we constructed a simulated sample to obtain training datasets in the absence of real annotated data. Specifically, we inserted all known Alu, SVA, and L1 sequences provided by Dfam (Hubley, et al., 2016), as well as other random sequences, into the reference genome of version hs37d5. And run MEHunter on the sample until it outputs consensus. Finally, we obtained the annotated Consensus dataset and fine-tuned DNABERT2 based on it.

Besides, we designed a loss function for the fact that some sequences are too long to be directly input into DNABERT2 for the high memory cost, therefore it is necessary to split the long sequence into pieces. For example, a 6k-bp L1 sequence will be split into 6 1k-bp pieces and then be calculated separately. However, certain parts aren't unique to one category, such as tandem repeats existing in both SVA and short tandem repeats (STR), therefore it's naturally necessary to lessen impacts of those parts on the final output. The details are as below.

First, the inputs and the whole model's outputs are represented as

$$S = \{ S_1, S_2, S_3, S_4, \ldots, S_n\} \quad |S_i|_{i<n} = L, |S_n| \leq L \quad (5)$$

$$Y^M_{output} = Y^M(S) = \{ Y^M_{S_1}, Y^M_{S_2}, Y^M_{S_3}, Y^M_{S4}, \ldots\ldots, Y^M_{S_n}\} \quad (6)$$

where S, Y^M represents inputs and model's outputs respectively, then, sort $Y^M{}_{output}$ based on Cross Entropy and get

$$Y^M_{sort} = \{ Y^M_{S_{t_1}}, Y^M_{S_{t_2}}, Y^M_{S_{t_3}}, Y^M_{S_{t_4}}, \ldots\ldots, Y^M_{S_{t_n}}\}, Sortkey(Y^M_{S_k}) = CrossEntropy(Y^M_{S_k}, Y_{answer}) \quad (7)$$

where Y_{answer} represents the label of answer. Finally, add heuristic constant modifications.

$$Y^M_{final} = Y^M_{S_{t_1}} + \frac{1}{e} Y^M_{S_{t_2}} + \frac{1}{e^2} Y^M_{S_{t_3}} + \frac{1}{e^3} Y^M_{S_{t_4}} + \ldots + \frac{1}{e^{n-1}} Y^M_{S_{t_n}} = \sum_{i=1}^{n} \frac{1}{e^{i-1}} Y^M_{S_{t_i}} \quad (8)$$

The intuitive explanation is that the parts that contradict the answer should be focused on learning, the parts that match the answer should avoid overfitting caused by excessive learning, and the ambiguous parts should be studied with caution. These three parts are located in the first half, second half, and middle of Y^{M}_{final}, respectively. By adding exponential decreasing constants to each term, the derivative decreases, and then the learning rate decreases.

2. Implementation of Benchmarking

2.1 Implementation of simulated data

The construction of simulated data involves three primary steps as follows:

Firstly, the construction of a reference genome. To ensure a substantial amount of precise Mobile Element (ME) insertions, we uniformly inserted 20,000 known Alu, SVA, and L1 sequences from Dfam across chromosomes 1-10 of the reference genome (version: hs37d5). These insertions included an equal distribution of Alu, SVA, and L1 sequences. Additionally, we inserted 5000 random, non-overlapping sequences with MEs using VISOR (Bolognini, et al., 2020).

Secondly, the generation of variant samples and simulated data. For the first haplotype, we uniformly inserted 20,000 Alu, SVA, L1, and 5000 random sequences onto chromosomes 11-20 of our constructed reference genome. This served to mimic both Mobile Element Insertion (MEI) and regular insertion events. The distribution of Alu, SVA, and L1 sequences keeps uniform. Then, on chromosomes 1-10, we deleted all sequences inserted in the first step to simulate Mobile Element Deletion (MED) and standard deletions. For the second haplotype, we selected half of the variants from the first haplotype to imitate ‘0/1’ and ‘1/1’ genotypes.

Finally, we employed lrsim (accessible at https://github.com/CoREse/lrsim), a versatile simulator compatible with any sequencing platform, to produce long-read sequencing data. This data was then processed using minimap2 and samtools (Li, et al., 2009) to yield the ultimate alignment data. Finally, we have constructed four ONT datasets (5×, 10×, 20×, 40×, respectively) and four PacBio HiFi datasets (5×, 10×, 20×, 40×, respectively).

Refer to subsection 2.3 for the command lines used for the generation of simulated datasets.

2.2 Evaluation of MEV callsets

The ME calls from simulated data were assessed based on the ground truths in the following approach. For ME insertions and deletions, if and only if the following conditions are met, a prediction is regarded as a true-positive (TP):

$$\begin{cases} max(\,comp_s - 1kbp, base_s) \leq min(\,comp_e + 1kbp, base_e) \\ min(\,comp_L, base_L)/\,max(\,comp_L, base_L) \geq 0.7 \\ comp_t = base_t \end{cases} \quad (9)$$

where $comp_s$, $comp_e$, $comp_L$, and $comp_t$ indicate start coordinate, stop coordinate, size, and SV class of a prediction, and $base_s$, $base_e$, $base_L$, and $base_t$ are starting coordinate, end coordinate, size, and SV class of a SV recorded in the ground truth, respectively. In addition, false positive (FP) refers to predictions that cannot meet the above conditions with any item in ground truths. Similarly, a ground truth MEV is determined as a false negative (FN) if and only if there is no ME call satisfies the above conditions with it. Then, based on the above concepts and definitions, precision is defined as

$$precision = \frac{TPs}{TPs + FPs} \tag{10}$$

Corresponding, recall is defined as

$$recall = \frac{TPs}{TPs + FNs} \tag{11}$$

F1-score is defined as

$$F1 = \frac{2 \times precision \times recall}{precision + recall} \tag{12}$$

and False discovery rate (FDR) is defined as

$$FDR = \frac{FPs}{TPs + FPs} \tag{13}$$

Furthermore, if taking the genotype of variation into consideration, a TP will be determined as TP-GT if and only if it has the same genotype as the corresponding ground truth ME, otherwise it will be recognized as FP-GT. Then, we can reuse equations above to calculate the statistics precision-GT, recall-GT and F1-GT under genotyping.

For the trio sample HG00731, HG00732 and HG00733, We used MDR (Mendelian Discordance Rate) index to demonstrate the logic and rationality of MEHunter's callset in parent-child transmission, where MDR is defined as

$$MDR = \frac{\sum offspring\text{'s } MEs \text{ not identified in parants}}{\sum offspring\text{'s } MEs} \tag{14}$$

To evaluate the computational performance of the MEV callers with different CPU threads/processes, CPU usage and memory overhead were recorded and assessed by using a python package called psutil. It is worth mentioning that if a main process forks out multiple child processes to complete computational tasks, the memory overhead is considered to be the sum of the Proportional Set Size (PSS) of all processes, rather than the Resident Set Size (RSS) of parent process itself. For cpu usage, it is similar. The tests were conducted on a platform with an AMD Ryzen 9 3950X, 128GB of DDR4 RAM dual channel at 4800 MHz, and Ubuntu 22.04.3 LTS.

Refer to subsection 2.3 for the command lines used for benchmarking MEHunter and rMETL.

2.3 The command lines for benchmarking

Contruction of reference genome with VISOR HACk (version 1.1.2)

```
VISOR HACk -b ./ins.bed -g HS37D5_DIR -o OUTPUT
```

Construction of variant genome sample with VISOR HACk (version 1.1.2)

```
VISOR HACk -b ./var.bed -g OUTPUT -o H1_DIR
VISOR HACk -b ./var2.bed -g OUTPUT -o H2_DIR
```

Pacbio HiFi data simulation with lrsim (v 0.5), samtools(v 1.11)and minimap2 (v 2.24-r1122):

1) reads simulation

```
export depth=(5, 10, 20, 30)
export sample_id={1, 2, 3, 4}
export threads=8
```

```
/mnt/mybook/lrsim2/lrsim -t $threads -s $sample_id \
 -d {2.5, 5, 10, 15} -e 0.001 \
 -m MODEL_PATTERN \
 H1/h1.fa H2/h1.fa > HiFi/reads_${sample_id}.fq \
 2> HiFi/log_${sample_id}.log
```

2) align the read

```
minimap2 -t $threads -a ../construct/output/h1.fa HiFi/reads_${sample_id}.fq > HiFi/SIM_HiFi_${depth}x_s${sample_id}.sam
samtools view -@ $threads -S HiFi/SIM_HiFi_${depth}x_s${sample_id}.sam -b > HiFi/SIM_FONT_${depth}x_s${sample_id}.bam
samtools sort -l 4 -@ $threads -o HiFi/SIM_HiFi_${depth}x_s${sample_id}_sort.bam HiFi/SIM_HiFi_${depth}x_s${sample_id}.bam
samtools index -@ $threads HiFi/SIM_HiFi_${depth}x_s${sample_id}_sort.bam
```

Pacbio CLR data simulation with lrsim (v 0.5), samtools(v 1.11)and minimap2 (v 2.24-r1122):

1) reads simulation

```
export depth={5, 10, 20, 30}
export sample_id={1, 2, 3, 4}
export threads=8
/mnt/mybook/lrsim2/lrsim -t $threads -s $sample_id \
 -d {2.5, 5, 10, 15} -e 0.10 \
 -m MODEL_PATTERN \
 H1/h1.fa H2/h1.fa > CLR/reads_${sample_id}.fq \
 2> CLR/log_${sample_id}.log
```

2) align the read

```
minimap2 -t $threads -a ../construct/output/h1.fa CLR/reads_${sample_id}.fq > CLR/SIM_CLR_${depth}x_s${sample_id}.sam
samtools view -@ $threads -S CLR/SIM_CLR_${depth}x_s${sample_id}.sam -b > CLR/SIM_CLR_${depth}x_s${sample_id}.bam
samtools sort -l 4 -@ $threads -o CLR/SIM_CLR_${depth}x_s${sample_id}_sort.bam CLR/SIM_CLR_${depth}x_s${sample_id}.bam
samtools index -@ $threads CLR/SIM_CLR_${depth}x_s${sample_id}_sort.bam
```

ONT data simulation with lrsim (v 0.5), samtools(v 1.11)and minimap2 (v 2.24-r1122):

1) reads simulation

```
export depth={5, 10, 20, 30}
export sample_id={1, 2, 3, 4}
export threads=8
/mnt/mybook/lrsim2/lrsim -t $threads -s $sample_id \
 -d {2.5, 5, 10, 15} -e 0.05 \
```

```
-m MODEL_PATTERN \
H1/h1.fa H2/h1.fa > ONT/reads_${sample_id}.fq \
2> ONT/log_${sample_id}.log
```

2) align the read

```
minimap2 -t $threads -a ../construct/output/h1.fa ONT/reads_${sample_id}.fq > ONT/SIM_ONT_${depth}x_s${sample_id}.sam
samtools view -@ $threads -S ONT/SIM_ONT_${depth}x_s${sample_id}.sam -b > ONT/SIM_FONT_${depth}x_s${sample_id}.bam
samtools sort -l 4 -@ $threads -o ONT/SIM_ONT_${depth}x_s${sample_id}_sort.bam ONT/SIM_ONT_${depth}x_s${sample_id}.bam
samtools index -@ $threads ONT/SIM_ONT_${depth}x_s${sample_id}_sort.bam
```

Benchmarking for MEHunter

1) cuteSV, signatures extraction.

```
conda activate cuteSVenv
cuteSV BAM REFERENCE OUTPUT_DIR --genotype WORK_DIR -s {1,2,3,5} -t 16 -L 10000 --report_readid --retain_work_dir --diff_ratio_merging_INS 1.1 --diff_ratio_merging_DEL 1.1 --diff_ratio_filtering_TRA 1.1
```

2) MEHunter calling.

```
conda activate MEHunter
MEHunter OUTPUT_DIR BAM WORK_DIR REFERENCE KNOWN_ME_DIR MEH_WORK_DIR MEH_OUTPUT_DIR --DL_module DL_module --retain_work_dir -t 16 --batch_size 4
```

Benchmarking for rMETL

1) Inference of putative MEI loci:

```
rMETL detection BAM REFERENCE WORK_DIR OUTPUT_DIR -t 16 -s {1, 2, 3, 5}
```

2) Realignment of chimeric read parts:

```
rMETL realignment PUTATIVE_LOCI ME_REFERENCE OUTPUT_DIR  -t 16
```

3) Mobile element insertion calling:

```
rMETL calling PUTATIVE_CLUSTER REFERENCE vcf OUTPUT_DIR
```

Benchmarking for xTea

1) Run.sh script (ONT, for example):

```
SAMPLE_ID=/mnt/mybook/xTeaReal/ONT/sample_ids.txt
BAMS=/mnt/mybook/xTeaReal/ONT/sample_bams.txt
WFOLDER=/mnt/mybook/xTeaReal/ONT/xTeaWork/
OUT_SCRTP=submit_jobs.sh
TIME=60:00
REF=/mnt/mybook/Real_data/hg38.fa
XTEA=/home/zzj/xTea-xTea_long_release_v0.1.0/xtea_long/
RMSK=/mnt/mybook/rep_lib_annotation/LINE/hg38/hg38_L1_larger_500_with_a
```

```
ll_L1HS.out
    CNS_L1=/mnt/mybook/rep_lib_annotation/consensus/LINE1.fa
    REP_LIB=/mnt/mybook/rep_lib_annotation/

    python ${XTEA}"gnrt_pipeline_local_long_read_v38.py"  -i ${SAMPLE_ID} \
    -b ${BAMS} -p ${WFOLDER} -o ${OUT_SCRTP} --xtea ${XTEA} -n 4 -m 12 \
    -t ${TIME} -r ${REF} --rmsk ${RMSK} --cns ${CNS_L1} --rep ${REP_LIB} \
    --min 4000  -f 31 -y 11 --clean --fast
```

2) run pipeline script:

```
    export PATH=$PATH:/home/zzj/bwa-master/
    export PATH=$PATH:/home/zzj/wtdbg2-master/
    PREFIX=/mnt/mybook/xTeaReal/HiFi5x/xTeaDir/HG00731/
    ############
    ############
    REF=/mnt/mybook/Real_data/hg38.fa
    XTEA_PATH=/home/zzj/xTea-xTea_long_release_v0.1.0/xtea_long/
    BAM_LIST=${PREFIX}"bam_list.txt"
    TMP=${PREFIX}"tmp/"
    REP_LIB=/mnt/mybook/
    SVA_REF_COPY=null
    ############
    ############
    python ${XTEA_PATH}"l_main.py" -C -b ${BAM_LIST} -r ${REF} -p ${TMP} -o ${PREFIX}"candidate_list_from_clip.txt"  -n 16 -w 75
    python ${XTEA_PATH}"l_main.py" -A -b ${BAM_LIST} -r ${REF} -p ${TMP} -i ${PREFIX}"candidate_list_from_clip.txt" -o ${PREFIX}"all_ins_seqs.fa" --rep ${REP_LIB} -n 16
    python ${XTEA_PATH}"l_main.py" -N -b ${BAM_LIST} -r ${REF} -p ${TMP}"ghost" -o ${PREFIX}"ghost_reads.fa" --rmsk /mnt/mybook/LINE/hg38/hg38_L1_larger_500_with_all_L1HS.out --cns /mnt/mybook/consensus/LINE1.fa --min 4000 -n 16
    python ${XTEA_PATH}"l_main.py" -Y -i ${PREFIX}"all_ins_seqs.fa" -r ${REF} -p ${TMP}"classification" --rep ${REP_LIB} -y 15 -o ${PREFIX}"classified_results.txt" -n 16
    python ${XTEA_PATH}"l_main.py" --clean -b ${BAM_LIST} -r ${REF} -p ${TMP} -i ${PREFIX}"candidate_list_from_clip.txt"  -n 16
```

Benchmarking for Palmer2

```
export PATH=$PATH:/mnt/mybook/ncbi-blast-2.10.0+/bin/
```

1) detecting Alu

```
rm -rf ./PALMER_5x_Alu
```

```
mkdir ./PALMER_5x_Alu

/mnt/mybook/PALMER/PALMER \
 --input
/mnt/mybook/Simulated/sample_genome/FCCS_UL/SIM_FCCS_UL_5x_s1_sort.bam \
 --workdir ./PALMER_5x_Alu/ \
 --ref_ver other \
 --ref_fa /mnt/mybook/Simulated/construct/output/h1.fa \
 --type ALU \
 --mode raw \
 --output PALMER_5x_Alu
```

2) detecting SVA

```
rm -rf ./PALMER_5x_SVA
mkdir ./PALMER_5x_SVA

/mnt/mybook/PALMER/PALMER \
 --input
/mnt/mybook/Simulated/sample_genome/FCCS_UL/SIM_FCCS_UL_5x_s1_sort.bam \
 --workdir ./PALMER_5x_SVA/ \
 --ref_ver other \
 --ref_fa /mnt/mybook/Simulated/construct/output/h1.fa \
 --type SVA \
 --mode raw \
 --output PALMER_5x_SVA
```

3) detecting LINE

```
rm -rf ./PALMER_5x_LINE
mkdir ./PALMER_5x_LINE

/mnt/mybook/PALMER/PALMER \
 --input
/mnt/mybook/Simulated/sample_genome/FCCS_UL/SIM_FCCS_UL_5x_s1_sort.bam \
 --workdir ./PALMER_5x_LINE/ \
 --ref_ver other \
 --ref_fa /mnt/mybook/Simulated/construct/output/h1.fa \
 --type LINE \
 --mode raw \
 --output PALMER_5x_LINE
```

References

Bolognini, D., *et al.* VISOR: a versatile haplotype-aware structural variant simulator for short- and long-read sequencing. *Bioinformatics* 2020;36(4):1267-1269.

Chen, Y., *et al.* Deciphering the exact breakpoints of structural variations using long sequencing reads with DeBreak. *Nat Commun* 2023;14(1).

Gao, Y., *et al.* abPOA: an SIMD-based C library for fast partial order alignment using adaptive band (vol 37, pg 2209, 2021). *Bioinformatics* 2021;37(19):3384-3384.

Hubley, R., *et al.* The Dfam database of repetitive DNA families. *Nucleic Acids Res* 2016;44(D1):D81-D89.

Jiang, T., *et al.* Long-read-based human genomic structural variation detection with cuteSV. *Genome Biol* 2020;21(1).

Li, H. Minimap2: pairwise alignment for nucleotide sequences. *Bioinformatics* 2018;34(18):3094-3100.

Li, H., *et al.* The Sequence Alignment/Map format and SAMtools. *Bioinformatics* 2009;25(16):2078-2079.

Ligowski, L. and Rudnicki, W.R. An efficient implementation of Smith Waterman algorithm on GPU using CUDA, for massively parallel scanning of sequence databases. *2009 IEEE International Symposium on Parallel & Distributed Processing* 2009:1-8.

Zhou, Z., *et al.* DNABERT-2: Efficient Foundation Model and Benchmark For Multi-Species Genome. In.; 2023. p. arXiv:2306.15006.
